# Stage IV gastric cancer successfully treated by multidisciplinary therapy including chemotherapy, immunotherapy, and surgery: a case report

**DOI:** 10.1186/s40792-017-0380-5

**Published:** 2017-10-23

**Authors:** Makoto Kawamoto, Hideya Onishi, Norihiro Koya, Hiroyuki Konomi, Kenji Mitsugi, Risa Tanaka, Junichi Motoshita, Takashi Morisaki, Masafumi Nakamura

**Affiliations:** 1 Department of Cancer Therapy and Research, Graduate School of Medical Sciences, Kyushu University, Fukuoka, Japan; 2 Fukuoka General Cancer Clinic, 3-1-1 Sumiyoshi, Hakata-ku, Fukuoka, 812-0018 Japan; 3 Department of Surgery, Hamanomachi Hospital, Fukuoka, Japan; 4 Department of Medical Oncology, Hamanomachi Hospital, Fukuoka, Japan; 5 Department of Pathology, Hamanomachi Hospital, Fukuoka, Japan; 6 Department of Surgery and Oncology, Graduate School of Medical Sciences, Kyushu University, Fukuoka, Japan

**Keywords:** Immunotherapy, Cytokine-activated killer cell, NKG2D, Gastric cancer, Dendritic cell

## Abstract

**Background:**

The prognosis of stage IV gastric cancer (GC) still remains unfavorable. Multidisciplinary approaches should therefore be considered to improve the survival of patients with stage IV GC. We report here a case of primary GC with potentially unresectable metastasis, successfully treated by a multidisciplinary approach including chemotherapy, immunotherapy, and surgery.

**Case presentation:**

A 74-year-old man presented with multiple left neck masses. Abdominal computed tomography showed a thickened gastric wall and multiple lymphadenopathies including left supraclavicular lymph node. Gastroenterological endoscopy revealed tumor lesions in the gastric cardia. Tumor biopsy indicated a pathological diagnosis of poorly differentiated adenocarcinoma. Open left cervical lymph node biopsy showed histological features identical with the gastric tumor, indicating left clavicle lymph node metastasis of GC. After 2 years of chemo-immunotherapy with S-1/CDDP, paclitaxel, and cytokine-activated killer cells, lesions other than the stomach lesion had regressed to undetectable on imaging studies. The patient then underwent laparoscopy-assisted total gastrectomy with Roux-en-Y reconstruction followed by adjuvant chemo-immunotherapy with paclitaxel and S-1 for 1 year, and immunotherapy with tumor lysate-pulsed dendritic cell-activated killer cells for 5 years. The patient remained well after 5 years and 6 months of follow-up, with no signs of recurrence.

**Conclusion:**

Therapeutic combinations including immunotherapy may thus allow surgery to be performed in patients previously considered unsuitable for surgical intervention, potentially leading to a clinical cure, as in the current case.

## Background

Gastric cancer (GC) is the second leading cause of cancer-related deaths worldwide [1]. Although systematic medical checks and the development of surgical treatments have contributed to the early detection and improved prognosis of GC, the median survival time of stage IV gastric cancer remains only 10–12 months [2].

Neo-adjuvant and adjuvant chemotherapies have been shown to improve overall survival in patients with far-advanced GC, but the rate of complete remission or long-term survival remains low [3, 4]. Immunotherapy involving adoptive transfer of activated killer lymphocytes has demonstrated survival benefits in advanced cancer [5]. We previously showed roles for immunotherapeutic approaches such as cytokine-activated killer (CAK) cell therapy, tumor-pulsed dendritic cell (DC) vaccine therapy, and DC-activated killer cell therapy in the treatment of patients with far-advanced cancer, either alone or combined with chemotherapy [6–9].

## Case presentation

**Fig. 1:**
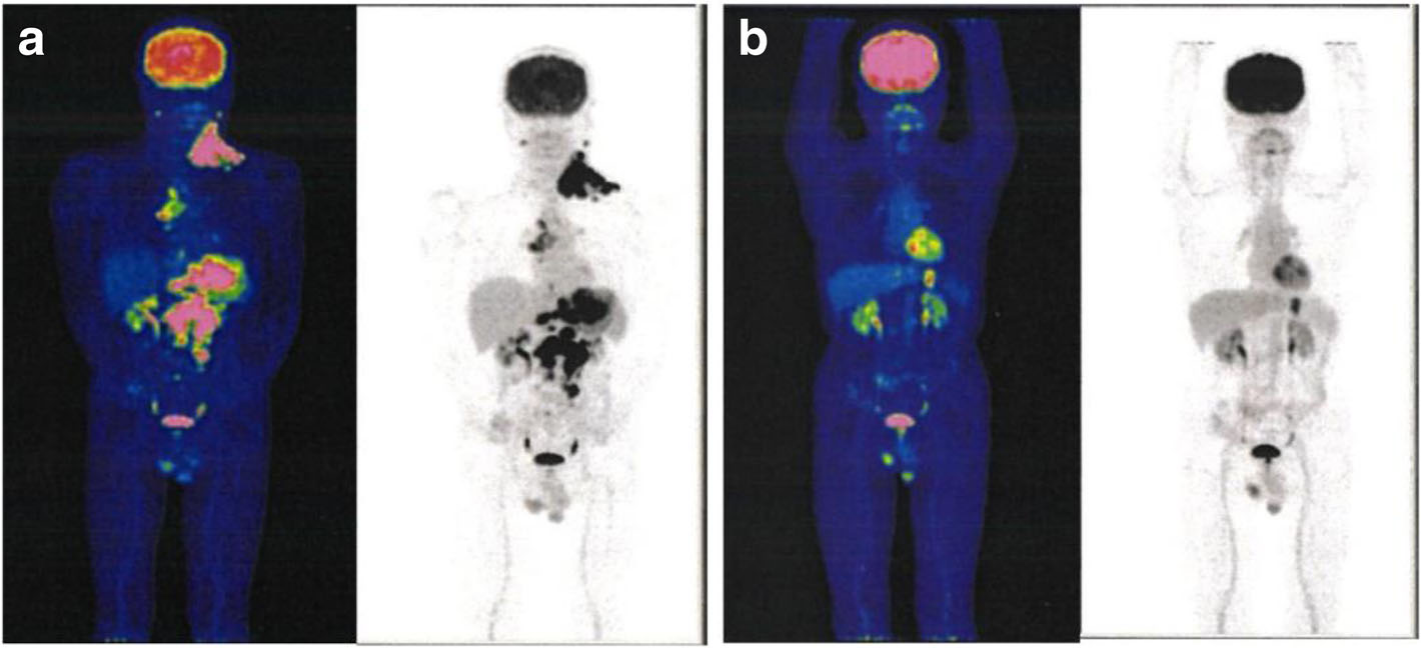
^18^F-fluorodeoxyglucose positron emission tomography findings. ^18^F-fluorodeoxyglucose positron emission tomography prior to treatment **a** in October 2009 and **b** in June 2011, following 2 years of chemo-immunotherapy. The metabolic values of the deposits in the lesions (such as left supraclavicular lymph nodes) other than the stomach decreased markedly or disappeared. The deposit in the stomach lesion and the sizes of all the lesions decreased markedly, compared with the sizes prior to treatment

**Fig. 2:**
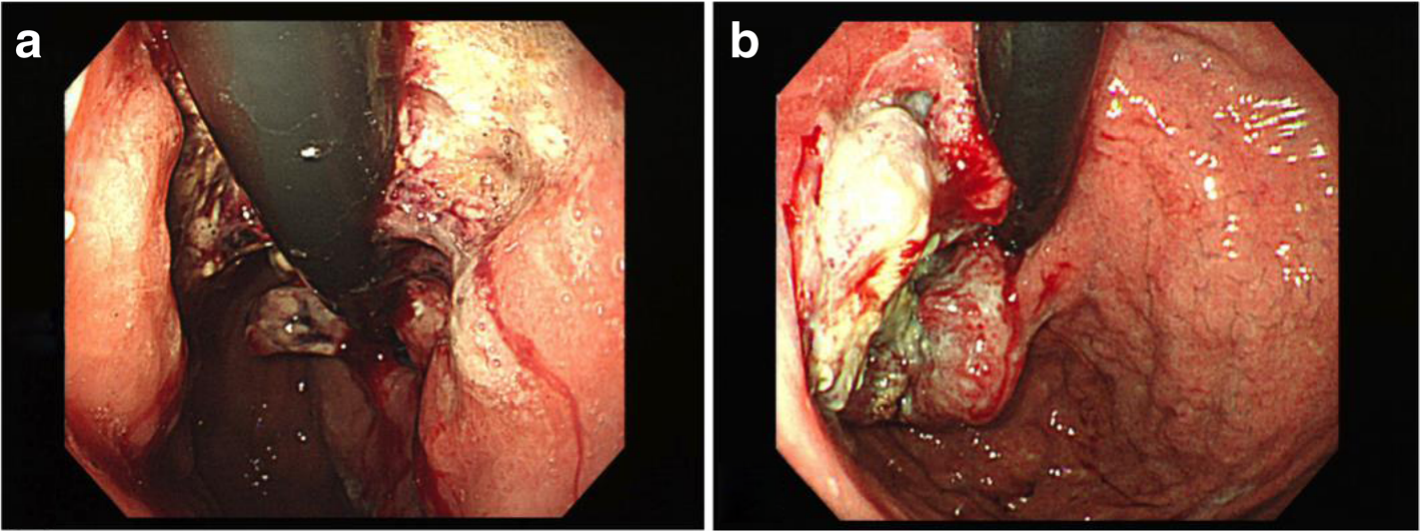
Gastroenterological endoscopy findings. Gastroenterological endoscopy revealed tumor lesion with widespread ulceration in the gastric cardia, **a** in October 2009 and **b** in June 2011. The stomach lesion decreased after 2 years of chemo-immunotherapy, preoperatively

A 74-year-old Japanese man was referred to our hospital with multiple left neck masses, a history of weight loss, and a slight fever. His body mass index was 18 kg/m^2^. He had an approximately 2-cm palpable but painless mass in his left neck. An abdominal mass and enlarged para-aortic lymph node were detected by abdominal ultrasonography. Laboratory testing showed serum C-reactive protein 2.5 mg/dl, serum hemoglobin concentration 5.8 g/dl, serum protein 5.9 g/dl, and serum albumin 2.7 g/dl, suggesting malnutrition and anemia caused by inflammation or bleeding. Carcinoembryonic antigen (CEA) was 31.6 ng/ml. The patient was admitted for further examination. Abdominal computed tomography (CT) scans showed a thickened gastric wall and multiple lymphadenopathies around the para-aortic lesion, portal section, mediastinum, and left supraclavicular soft tissue. ^18^F-fluorodeoxyglucose positron emission tomography (FDG-PET) revealed abnormal FDG uptake in the same lesions detected by CT (Fig. 1a). Gastroenterological endoscopy revealed a tumor lesion with widespread ulceration in the gastric cardia, with lower esophageal invasion (Fig. 2a). A biopsy of the tumor lesion indicated a pathological diagnosis of poorly differentiated adenocarcinoma. Open left cervical lymph node biopsy showed histological features identical with the gastric tumor, indicating left clavicle lymph node metastasis of GC. Based on these clinical and pathological findings, the clinical stage was T4aN3bM1, stage IV, according to the Union for International Cancer Control (UICC 7th edition).

In consideration of his progressive disease, the patient received a blood transfusion for chemotherapy and S-1 plus cisplatin (S-1/CDDP) was then initiated at Hamanomachi Hospital. S-1 (100 mg/day) was administered orally for three consecutive weeks, and cisplatin (90 mg/body) was administered intravenously on day 8, followed by a 2-week rest period. The left neck tumor and abdominal mass had regressed rapidly after first chemotherapy as well as CEA decrease, and we administered combined CAK cell therapy and S-1/CDDP at our clinic.

**Fig. 3:**
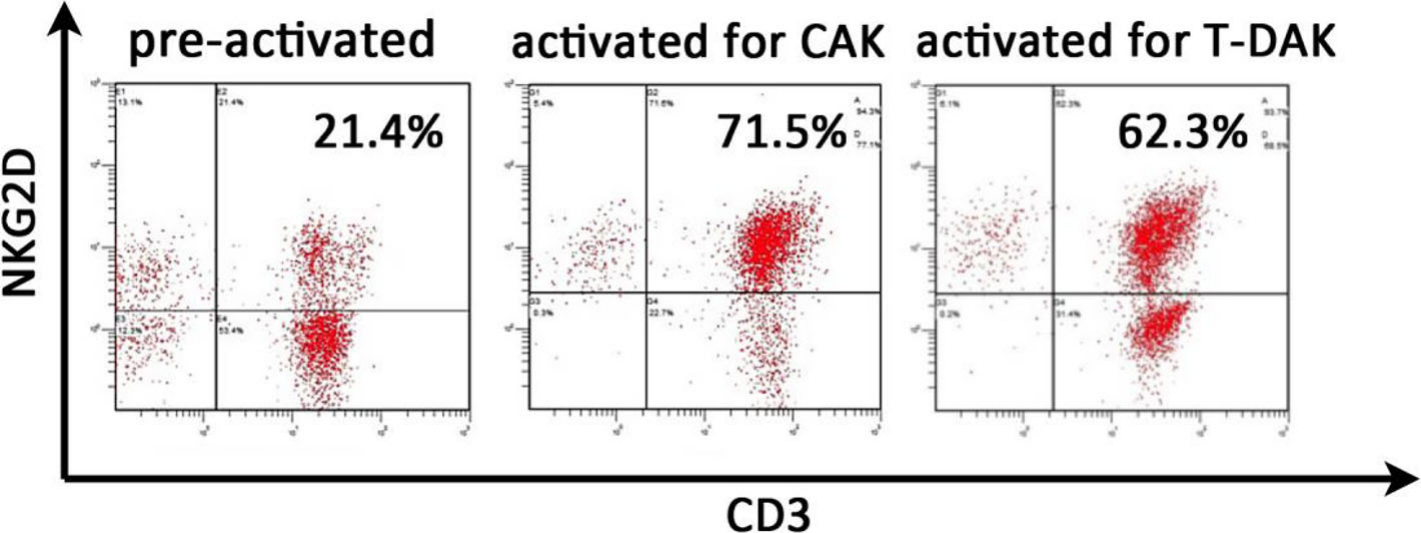
Representative histogram of natural-killer group 2, member D (NKG2D) expression on lymphocytes before and after stimulation. NKG2D expression levels on lymphocytes before and after activation were analyzed by fluorescence-activated cell sorting. Activation resulted in a notable increase in NKG2D expression on CAK and T-DAK cells

**Fig. 4:**
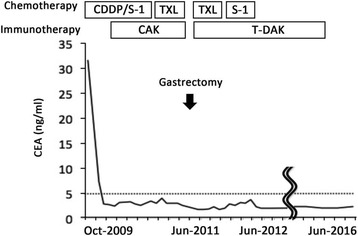
Decrease in CEA levels throughout the whole treatment course

**Fig. 5:**
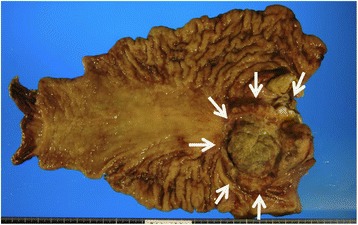
Resected specimen of stomach showing a Borrmann II type tumor with elevated lesion in the posterior wall of the cardia (arrows)

**Fig. 6:**
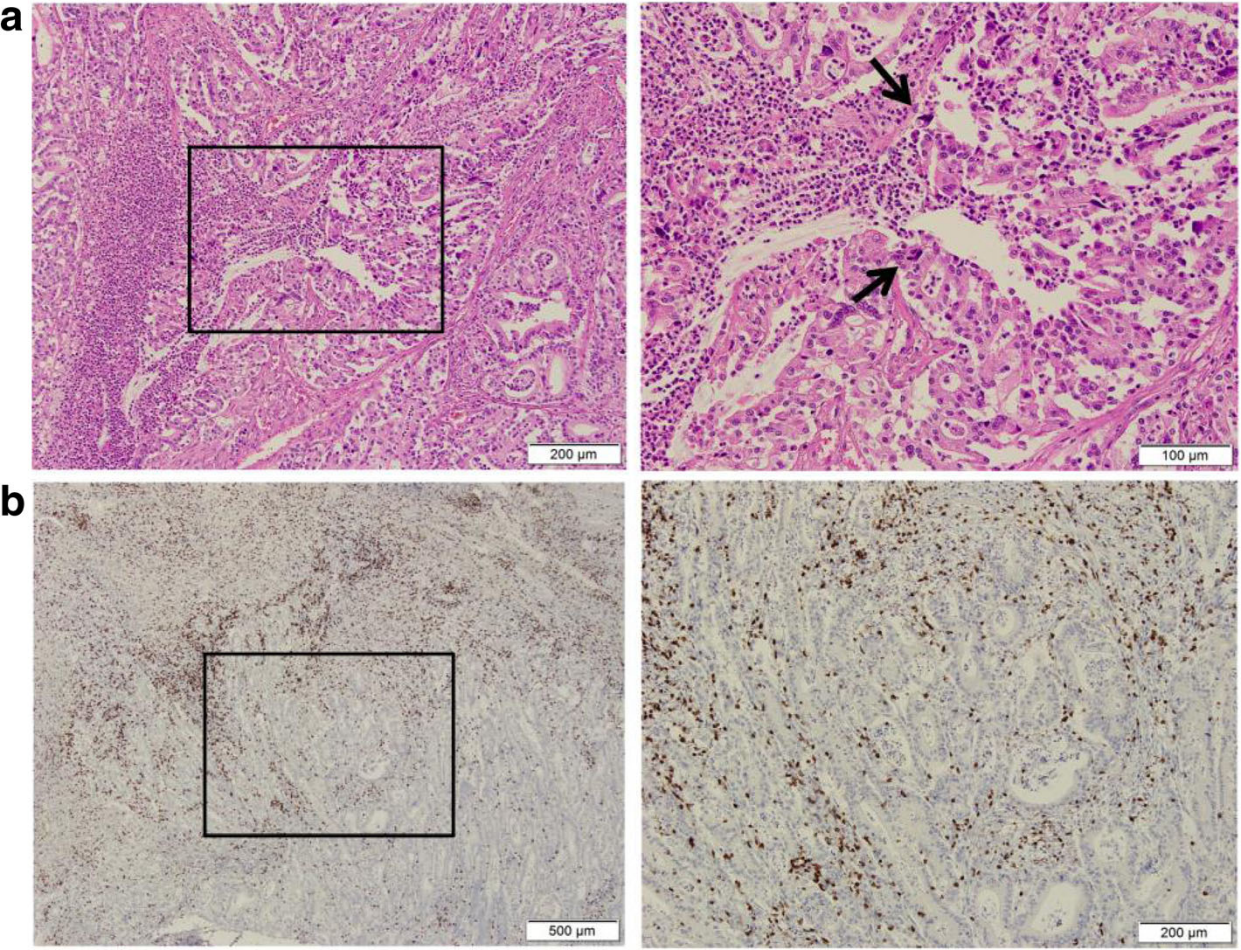
Representative hematoxylin–eosin-stained images and CD3+ immunohistochemistry results in the resected specimen. **a** Tumor with irregular pyknotic nuclei (arrows) and tumor-infiltrating lymphocytes. Original magnification ×200 (right) and corresponding (boxed) areas with lower magnification ×100 (left). **b** Rich lymphocytic infiltration into the tumor. CD3^+^ T cells are indicated by the brown chromogen. Original magnification ×100 (right) and corresponding (boxed) areas with lower magnification ×40 (left)

CAK cells consisted of activated T cells expressing high levels of the activating receptor, natural-killer group 2, member D (NKG2D), and activated natural killer (NK) cells (Fig. 3). The procedure for CAK cell generation has been described previously [8]. Briefly, peripheral blood mononuclear cells (PBMCs) were collected using a blood cell separator (Haemonetics CCS, Haemonetics Corporation, Braintree, MA, USA) and stimulated with human recombinant interleukin (IL)-2 (rIL2, 200 U/ml; Primmune Inc. Kobe, Japan) and 5 μg/ml antibody to CD3 (MACS GMP CD3 pure; Miltenyi Biotec Inc. Auburn, CA, USA). After 7 days in culture, PBMCs were transferred to a culture-bag system (KBM550, Kohjin Bio, Osaka, Japan) and expanded for 7 days to obtain sufficient numbers of CAK cells. All cultures were inspected for contamination with endotoxin, β-glucan, and peptide-glycan using a Toxinometer ET-6000 (Wako Pure Chemical Industries, Ltd., Osaka Japan), according to Food and Drug Administration guidelines. Mycoplasma contamination was checked using a Mycoalert kit (Lonza Rockland Inc., ME, USA). The cell processing and immunotherapy procedures were approved by the ethics committee of our institution, and the patient provided written informed consent for the procedure. A total of 1.32 × 10^10^ CAK cells were transferred 14 times over 2 years. After four courses of S-1/CDDP administration, the patient developed chemotherapy-related anorexia and stomatitis (Common Terminology Criteria for Adverse Events grade 3), and chemotherapy was switched to weekly paclitaxel at the request of the patient. Paclitaxel (120 mg/body) was administered weekly, three times every 4 weeks. CAK cell therapy was well tolerated, and the only treatment-related adverse event was low-grade fever. After 2 years of combined chemotherapy (S-1/CDDP, paclitaxel) and CAK cell immunotherapy, the lesions other than the stomach lesion had markedly regressed according to CT and FDG-PET (Fig. 1b), and CEA had decreased from 30.2 ng/ml to 2.2 ng/ml (Fig. 4). We confirmed the maximum reduction in the gastric lesion by gastroenterological endoscopy (Fig. 2b), prompting us to consider conversion surgery. Confirming peritoneal lavage cytology negative and no peritoneal dissemination or liver metastasis, we proceeded to laparoscopy-assisted total gastrectomy for GC with D1+ lymphadenectomy and Roux-en-Y reconstruction. The patient’s postoperative course was uneventful, and he was discharged home on postoperative day 18, tolerating an oral diet. The histological findings of the resected specimen (Fig. 5) showed tumor invasion into the subserosal layer and only one lymph node metastasis in 46 dissected lymph nodes. The resected specimen showed the emergence of irregular pyknotic nuclei and many infiltrating mononuclear CD3+ cells by immunohistochemistry (Fig. 6a, b). These findings suggest the effects of chemotherapy and immunotherapy, respectively. Accumulation of form cells was detected in some dissected lymph nodes, suggesting regression of the metastatic lesions. Based on clinical and these pathological findings, the postoperative stage was ypT2ypN1cM0, ypStage IIA, according to the Union for International Cancer Control (UICC 7th edition).

The patient was treated with adjuvant chemotherapy consisting of paclitaxel plus S-1 for 1 year and immunotherapy with tumor lysate-pulsed DC-activated killer (T-DAK) cell immunotherapy for 5 years. Paclitaxel (120 mg/body) was administered weekly, twice every 4 weeks for four courses, after which it was replaced by S-1 because of difficulty in securing vascular access. S-1 (80 mg/day) was administered orally for 2 weeks, followed by a 2-week rest period. The methods used to prepare the T-DAK cells have been described previously [10]. Firstly, immature DCs were prepared from plastic-adherent PBMCs using recombinant human granulocyte/monocyte colony-stimulating factor (100 ng/ml; Primmune KK, Kobe, Japan) and recombinant human IL-4 (500 U/ml; Primmune KK) for 7 days. Tumor cells obtained from the tumor masses were lysed by five freeze–thaw cycles and then adjusted to 1 mg protein concentration. The tumor lysate was added to immature DCs at a final concentration of 50 μg/ml and then incubated for 12 h, followed by the addition of tumor necrosis factor and interferon-γ. Secondly, non-adherent cells among the patient’s PBMCs were cultured with autologous tumor-pulsed DCs for 1 week in low dose IL-2, then expanded in OKT3-coated flasks followed by culture in oxygen-permeable bags (KBM550BA-M; Kojin-Bio, Saitama, Japan). A total of 3.0 × 10^10^ T-DAK cells were infused 29 times over 5 years. The patient’s clinical tolerance during and after the adjuvant therapy was excellent, and no adverse reactions were observed. The above cell processing and immunotherapy procedures for T-DAK cell immunotherapy were approved by the ethics committee of our institution.

The patient was followed up carefully and remained well with no signs of recurrence at 5 years and 6 months after surgery.

## Discussion

Multidisciplinary approaches for stage IV GC have received considerable attention [11]. Immunotherapy may be included among these multidisciplinary methods in the case of far-advanced cancers. For instance, Kimura et al. reported a survival benefit associated with postoperative immunotherapy among patients with primary lung cancer undergoing non-curative resection, compared with patients receiving radiation therapy or chemotherapy [12]. Kimura et al. also reported that adjuvant immunotherapies improved the postsurgical prognosis in a randomized controlled phase III trial in patients with lung cancer [13]. Yang et al. reported that DC-activated cytokine-induced killer cells enhanced the anti-tumor effects of chemotherapy in patients with advanced non-small cell lung cancer [14]. Therapeutic combinations including immunotherapy may thus allow surgery to be performed in patients previously considered unsuitable for surgical intervention, potentially leading to a clinical cure, as in the current case.

Although chemotherapy can achieve tumor regression of acute phase disease, chemotherapy alone may be unable to control cancer progression completely because of drug resistance or the presence of cancer stem cells. In these cases, other treatments such as immunotherapy should be combined with chemotherapy to eliminate troublesome cancer cells. Immunotherapy may provide cancers with an immune surveillance mechanism, regardless of the presence of drug resistance or cancer stem cells [15, 16], which is indispensable for long-term anti-tumor efficacy. The numbers of tumor-infiltrating lymphocytes have been reported to be a prognostic factor [17]. We also previously reported on the synergistic effects of gemcitabine and CAK cell immunotherapy [18].

The CAK cells used in the current case were a heterogeneous population including activated NK cells and T cells, which are important effectors in the immune response to tumors in a tumor-antigen-independent fashion [18]. NKG2D is an activating receptor found on activated NK and activated T cells and was used to characterize the infused CAK and T-DAK cells. Previous reports showed that the strength of the anti-tumor immune response depended on the surface levels of NKG2D [19, 20]. CAK cells were thought to play an important role in tumor reduction in the current patient. CD3+ tumor-infiltrating lymphocytes detected in the resected specimen were considered to reflect this.

The T-DAK cells administered as postoperative immunotherapy in this patient are thought to have a potent tumor-specific cytotoxic effect, because autologous tumor lysate-pulsed DCs induce a wide array of T cell responses against both known and unknown tumor-specific antigens through MHC class-I-restriction [21]. However, a potential disadvantage of T-DAK cell immunotherapy includes autoimmune reactivity to normal tissue antigens as a result of the processing and presentation of housekeeping or lineage-associated epitopes by autologous DCs. In this patient, the only immunotherapy-related adverse event was low-grade fever. We inferred that the tumor-specific immune surveillance mechanism generated by T-DAK cell immunotherapy enabled the current patient to maintain continuous remission for over 5 years, despite the unresected initial metastatic lesions.

Staging laparoscopy has become a useful modality for determining unresectability or effects of preoperative therapy, given the advantage of avoiding unnecessary laparotomy [22]. In this patient, we first confirmed no unresectable diseases and proceeded to laparoscopic surgery, resulting in favorable short- and medium-term outcome. However, the surgical procedure for this patient may be controversial, considering not having established superiority in laparoscopic surgery for advanced GC and possibility of much information in conventional laparotomy. Surgical procedure for conversion surgery deserves further investigation.

Although it was not possible to distinguish between the contributions of chemotherapy and immunotherapy to the clinical response, the present case nevertheless suggests that chemo-immunotherapy can effectively suppress the progression of GC and may induce a long-term anti-tumor environment.

## Conclusions

We report here a case of primary GC with potentially unresectable metastasis, successfully treated by a multidisciplinary approach including chemotherapy, immunotherapy, and surgery. This approach thus deserves further investigation to confirm its value in patients with far-advanced cancers.

## Abbreviations

CAK: Cytokine-activated killer
CEA: Carcinoembryonic antigen
CT: Computed tomography
DC: Dendritic cell
FDG-PET: ^18^F-fluorodeoxyglucose positron emission tomography
GC: Gastric cancer
NK: Natural killer
NKG2D: Natural-killer group 2, member D
PBMCs: Peripheral blood mononuclear cells
T-DAK: Tumor lysate-pulsed DC-activated killer
